# Supplementary material for: CpG and UpA dinucleotides in both coding and non-coding regions of echovirus 7 inhibit replication initiation post-entry
Source: eLife. 2017 Sep 29;6:e29112. doi: 10.7554/eLife.29112 (PMC5659819; doi:10.7554/eLife.29112)
Supplement: Supplementary File 1. — (B) Probes for RNA fluorescent in situ hybridization. [file elife-29112-supp1.docx]

# Supplementary file 1A

**Oligonucleotides used in this study**

| **Name** | **Sequence 5’ to 3’** |
| --- | --- |
| PCR_7146s PCR_7358as PCR_7315s  PCR_7494as  E7 5’UTRs E7 5’UTRas  R2_WT_P_ApaI_Rev R2_C_ApaI_Rev R2_U_ApaI_Rev  R2_cu_ApaI_Rev R2_WT_EcoRI_Fwd R2_P_EcoRI_Fwd  R2_C_EcoRI_Fwd R2_U_EcoRI_Fwd  hGAPDH F hGAPDH R E7-GND_F  E7-GND_R | GGTGGACCAAAGACCCAAAGAACACTCAAG TTGTCTCTAATGTTAACCCTGCAGGGTCGACTTAAAAAGAGTCCAACCACTTCCTGCGC GGACTCTTTTTAAGTCGACCCTGCAGGGTTAACATTAGAGACAATTTAAACTAATTTG  GGGAACAAAAGCTGGAGCTCCGCGGCCGC  TCCGGCCCCTGAATGCGGCTAA CACCCAAAGTAGTCGGTTCCGC ACAGGGCCCAGATCTGAGTTCATCTTTCACGTATGTCACC ACAGGGCCCAGATCTGAGTTCGTCTTTCACGTACGTCACC  ACAGGGCCCAGATCTTAGTTCATCTTTAACGTATGTAACC ACAGGGCCCAGATCTGAGTTCATCTTTCACATATGTCACC ACTGGAATTCGCCGCCACCATGCCGTTGCTATGATGAAGAGAAACTCAAGTAC  ACTGGAATTCGCCGCCACCATGCAGTGGCTATGATGAAGAGAAACTCAAGCAC  ACTGGAATTCGCCGCCACCATGCGGTCGCGATGATGAAGCGTAACTCGAGTAC ACTGGAATTCGCCGCCACCATGCCGTAGCTATGATGAAGAGAAACTCTAGTAC GAAATCCCATCACCATCTTCCAGG  GAGCCCCAGCCTTCTCCATG GGATGATTGCATATGGTAACGATGTGATTGCGTCATAC GTATGACGCAATCACATCGTTACCATATGCAATCATCC |

# Supplementary file 1B

Probes for RNA fluorescent in situ hybridization

| # | Sequence |
| --- | --- |
| 1 | TTGATTTGATGTACGGGCTC |
| 2 | CAGGTTCGTGGGGTTTAAAT |
| 3 | GGGCACATATGTGATGGATG |
| 4 | GCACATCTGGACGTATAGTG |
| 5 | TAACTGCCAACATACACAGC |
| 6 | TAGGTGTCTATTGACCACTC |
| 7 | CACAGTTCTGCCAGTCATTG |
| 8 | AGGTCTCTGTTGTAGTCTTC |
| 9 | GGCAGTAGTTGTGCTCACAA |
| 10 | GCATCTGGCTATAATGTCAC |
| 11 | GACAGAAGTACACTCCTGTT |
| 12 | CCTCAAAGCTTACTGGGTAA |
| 13 | TAGTACTCACTTTCCTGGAC |
| 14 | ACATGGGATTGGTACCTTTT |
| 15 | TGAAAATCCTGCCGCCAACA |
| 16 | GAGTATCCCACCACA ATCAC |
| 17 | CAACGACACCGTGTTCACAT |
| 18 | TCACCACCCATAGTGACGAT |
| 19 | AGTCACGGATGTCTGCAAAG |
| 20 | GCATCATCCTCTAACCATAA |
| 21 | GTTCGACATAGTCCTTGATC |
| 22 | GAACCAAAGGCATTGCCGAG |
| 23 | GCTCACATATTTGATTGGTG |
| 24 | GTGACTCTTTCAAGAGGTTG |
| 25 | TAGAATGGAGTCTTGACCCA |
| 26 | GGTTTCTCACCACAATTACT |
| 27 | CAGTCACCGTGATTAAGTCA |
| 28 | ACAACCAATGAGGGCTAGTG |
| 29 | ACACTTTCTGTTTGAGCCAC |
| 30 | CCATGGGTATTCCGTAATAT |
| 31 | TAAGCCATCCGTTATTTTGG |
| 32 | GTTTGTCATCTCAGTGAACT |
